# Radiographic Outcomes of Percutaneous Pinning for Displaced Extra-Articular Fractures of the Distal Radius: A Time Course Study

**DOI:** 10.1155/2014/540874

**Published:** 2014-05-05

**Authors:** Tien-Yu Yang, Yao-Hung Tsai, Shih-Hsun Shen, Kuo-Chin Huang

**Affiliations:** ^1^Department of Orthopaedic Surgery, Chang Gung Memorial Hospital, 6 West Section, Chia-Pu Road, Pu-Tz City, Chiayi County 613, Taiwan; ^2^Chang Gung University College of Medicine, Taoyuan 333, Taiwan

## Abstract

*Introduction*. Although not all malunited distal radius fractures are symptomatic, the goal of treatment for displaced extra-articular fractures of the distal radius should be to restore and to maintain the radial geometry until bone healing. However, the time course change after surgery for these fractures is unclear. *Methods*. We, therefore, performed a retrospective cohort study on patients who sustained such fractures treated with percutaneous pinning. The main outcome measures in this study included four radiographic measurements: radial height, radial inclination, radial tilt, and ulnar variance. *Results*. Assessment of the monthly changes in these measurements revealed that early fracture collapse with loss of the reduced radial tilt occurred. Besides, among the 4 measurements, the normal radial tilt was the most difficult to be achieved when repositioning and pinning the fractured fragments. *Conclusions*. Even though the modified Kapandji technique provided a superior ability to maintain the reduced position until bone healing over the Willenegger method, we recommended that refinement of surgical techniques and postoperative hand care program may be necessary to fulfill the treatment objectives of stable surgical fixation and early joint motion.

## 1. Introduction

Malunion of a displaced extra-articular fracture of the distal radius may affect the mechanics of the radiocarpal joint, the distal radioulnar joint (DRUJ), and the forearm axis and then result in wrist pain, motion loss, and/or decreased grip strength [1–6]. Although not all malunited distal radius fractures are symptomatic, the goal of treatment for displaced extra-articular fractures of the distal radius should be to restore and to maintain the radial geometry until bone healing. For assessing healing and predicting outcome of a distal radius fracture, Graham [7] proposed and popularized the evaluation criteria based on four familiar radiographic measurements, including radial height (RH), radial inclination (RI), radial tilt (RT), and ulnar variance (UV). Today, Graham's criteria have become one of the most widely practiced guidelines for treatment of patients with distal radius fractures.

Clinical practice guidelines from the American Academy of Orthopaedic Surgeons (AAOS) moderately recommend stable surgical fixation, rather than cast fixation, followed by early wrist motion to treat patients with displaced distal radius fractures [8]. A key method of surgical fixation is percutaneous pinning, involving the insertion of Kirschner- (K-) wires through the skin to stabilize the fracture. Although there is some evidence to support its use, the precise role and methods of percutaneous pinning are not well established [9]. Among the various methods, Kapandji and conventional pinning (Willenegger) techniques are the two most commonly used methods to treat displaced and unstable distal radius fractures without significant intra-articular displacement [10–15]. The main concern in percutaneous pinning for fracture fixation is the reliability of maintenance of the reduced position [13, 14, 16, 17]; however, little is known about the time course changes and patterns of fracture collapse after percutaneous pinning for displaced extra-articular fractures of the distal radius. This study was thus designed to evaluate the effects of two commonly used pinning techniques on the achievement and maintenance of the reduced radial geometry after fracture reduction and pin fixation for displaced extra-articular fractures of the distal radius. Our objective was to explore the time course changes of fracture collapse in these patients.

## 2. Materials and Methods

### 2.1. Participating Patients and Inclusion/Exclusion Criteria

The study included all adult patients with displaced extra-articular fractures of the distal radius (AO/OTA type 23-A2.2 or 23-A3) who were treated with percutaneous pinning in our hospital between January 2006 and December 2009. The institutional review board of Chang Gung Memorial Hospital approved this study. We excluded patients with bilateral distal radius fractures, open or multiple fractures, underlying bone pathology, and medical problems that could severely impair bone healing and self-care ability, such as malignancies, osteomalacia, end stage renal diseases, and advanced neurodegenerative disorders.

### 2.2. Surgical Techniques

Two techniques of percutaneous pinning for displaced extra-articular fractures of the distal radius were regularly used in our hospital. One is the modified Kapandji technique; the other is the conventional pinning (Willenegger technique). For patients treated with the modified Kapandji technique, the reduction was performed with two intrafocal pins over a firm pad and checked under image intensification. A K-wire was inserted into the fracture site from the dorsal-ulnar side and then used to pry the distal fragment into optimal position to reestablish the palmar tilt. The other K-wire was inserted from the palmar-radial side to reestablish the RH and the RI. The K-wires were then levered until the acceptable anatomy was restored, and subsequently another two K-wires were inserted through stab incisions over the radial styloid process and the dorsal-ulnar corner of the radius in a conventional manner. The ends of the last two K-wires were bent over and left percutaneously, and the first two K-wires were removed to prevent skin indentation or tethering.

For those treated with the Willenegger technique, the reduction was facilitated and held with Chinese finger traps and checked under image intensification. After restoration of the acceptable anatomy, two small stab incisions were made over the radial styloid process and two K-wires were inserted obliquely through the styloid process from distal to proximal, with the proximal wire tip embedded and anchored in the opposite cortex of the radius. The ends of the two K-wires were bent over and left percutaneously.

Postoperative care was implemented according to a standardized regimen. The wrist was immobilized for 8 weeks in a palmar short-arm splint. In the meanwhile, active finger motion and forearm axis rotation were encouraged, but power grip even in the splint was avoided. For the next 4 weeks, physiotherapy focusing on active and passive motion exercises for the wrist and forearm was performed with the splint removed only for rehabilitation. Thereafter, the splint was discarded and power grip was permitted. Regular radiographic assessments were carried out after both procedures: they were done immediately after surgery; at one, two, three, six, and twelve months; and before and after removal of the K-wires. The K-wires were removed from all wrists, as outpatients, usually after six weeks.

### 2.3. Definition of Treatment Outcome

The main outcome measures in this study included four radiographic measurements: RH, RI, RT, and UV. RH, measured from the PA radiograph, is defined as the distance between two perpendiculars to the long axis of the radius, one drawn at the tip of the radial styloid process and the other at the ulnar corner of the lunate fossa, which averagely should be 11 to 12 mm. RI, measured from the PA radiograph, is defined as the angle between a line joining the tip of the radial styloid process and the ulnar corner of the lunate fossa and a line drawn perpendicular to the long axis of the radius, which averagely should be around 23°. RT, measured from the lateral film, is defined as the angle between a line joining the dorsal and palmar lips of the distal radial articular surface and a line drawn perpendicular to the long axis of the radius, which averagely should be around 11° (palmar tilt). UV, measured from the PA film, is defined as the distance between two perpendiculars to the long axis of the radius, one from the ulnar corner of the lunate fossa and the other from the distal ulnar articular surface, which should be ulnar neutral in 60% of the population [18].

### 2.4. Patient Characteristics and Study Significance

We summarized the collected data at the time of study enrollment, which included demographic data, fracture types based on the AO/OTA classification system, surgical techniques, monthly radiographic assessments, and treatment complications such as nonunion, complex regional pain syndrome (CRPS), infection, or loosening. These data were analyzed to study the time course changes and patterns of fracture collapse and the causal relationship between surgical techniques and treatment outcomes. Results of this analysis may provide clues for refinement of surgical techniques and postoperative hand care program in future studies.

### 2.5. Statistical Analysis

A chi-square analysis or a Fisher's exact test was used when appropriate for analyzing categorical variables. For numerical variables, the nonparametric Wilcoxon rank sum test was used for between-group comparisons. Statistical significance was defined as *P* < 0.05. All statistics were two-sided and performed using the Statistical Package for the Social Sciences (SPSS, v12.0; SPSS Inc., Chicago, Illinois).

## 3. Results

### 3.1. Analyses of Patient Characteristics and Related Variables

Eighty-five patients with displaced extra-articular fractures of the distal radius (AO/OTA type 23-A2.2 or 23-A3) were enrolled in this 4-year-long study. The mean patient age was 58.3 years (range 20–80); twenty (23.5%) were male, and 65 (76.5%) were female. 60% of fractures were the left wrist and 65% were type 23-A3. All patients had undergone the above-mentioned techniques of percutaneous pinning for fracture reduction and pin fixation. Twenty-five (29.4%) patients were treated with the modified Kapandji technique (group 1: Kapandji) and 60 treated with the Willenegger technique (group 2: Willenegger). Comparison of groups 1 and 2 revealed no significant differences in age, gender, involved lesion wrists, fracture types, and treatment complications such as nonunion, CRPS, infection, or loosening (all *P* ≥ 0.095) (Table 1). There were no tendon or vascular complications and no dysfunction of the median nerve in this cohort.

### 3.2. Analyses of Radiographic Measurements before and after Fracture Reduction

All fractures in this cohort were diagnosed as displaced, meaning the fractured fragments were out of normal alignment. The mean (±SD) RH, RI, RT, and UV values in groups 1 and 2 patients before fracture reduction were 9.63 (±2.84) mm versus 8.76 (±2.31) mm (*P* = 0.143), 20.00 (±5.37) degrees versus 18.63 (±4.43) degrees (*P* = 0.228), −17.64 (±12.81) degrees versus −19.01 (±9.81) degrees (*P* = 0.757), and 3.25 (±3.04) mm versus 3.74 (±2.24) mm (*P* = 0.617), respectively. Attempts to manipulate the fractured fragments back into acceptable alignment were performed through the above-mentioned surgical techniques. The mean (±SD) RH, RI, RT, and UV values in groups 1 and 2 patients after fracture reduction were 11.82 (±2.32) mm versus 12.13 (±1.80) mm (*P* = 0.510), 23.63 (±4.05) degrees versus 24.36 (±2.60) degrees (*P* = 0.323), 2.06 (±5.78) degrees versus 3.78 (±4.35) degrees (*P* = 0.136), and 1.36 (±2.70) mm versus 1.37 (±1.65) mm (*P* = 0.996), respectively. Although there were no significant differences in the radiographic measurements before/after fracture reduction between the two groups, the reduction technique used in group 2 patients produced a more anatomic reduction in RH (2.20 ± 2.32 mm versus 3.38 ± 2.06 mm, *P* = 0.010) and RI (3.64 ± 2.59 degrees versus 5.73 ± 3.86 degrees, *P* = 0.015) compared to that used in group 1 patients (other *P* ≥ 0.136) (Table 2).

### 3.3. Analyses of Radiographic Measurements after Fracture Fixation

**Figure 1:**
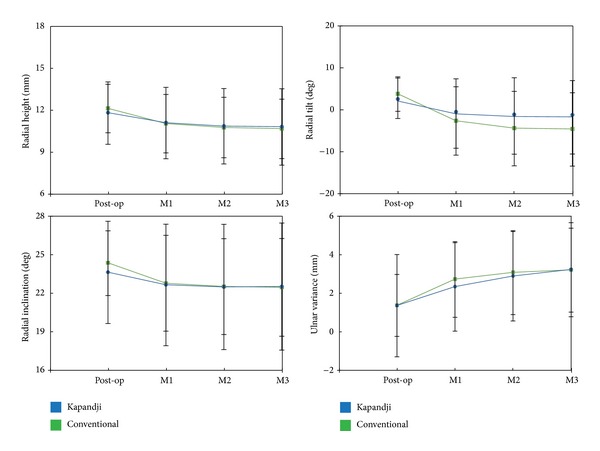
Group comparison of radiographic parameters after fracture fixation. M*x*:* x* months after the index surgery.

**Figure 2:**
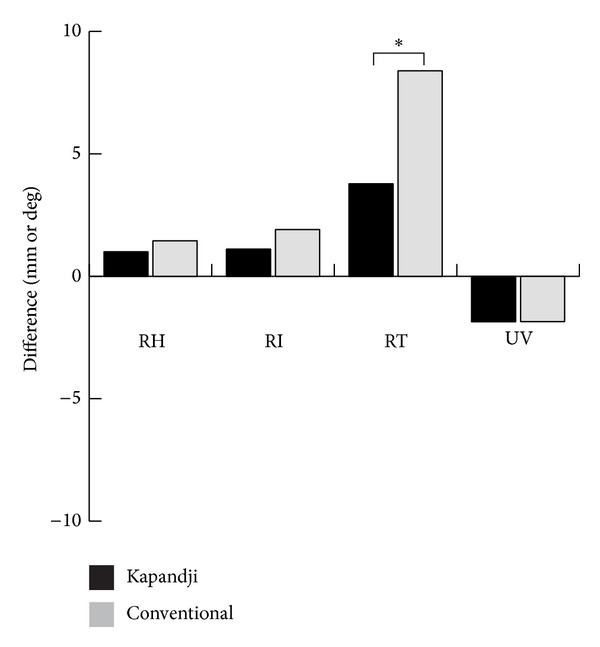
Group comparison of the final loss of alignment after fracture fixation. RH: radial height; RI: radial inclination; RT: radial tilt; UV: ulnar variance; *denotes that the difference is significant (*P* < 0.05) by Wilcoxon rank sum test.

In order to clarify the time course changes and patterns of fracture collapse after K-wire fixation in the two groups, we analyzed the radiographic measurements on the wrist films at follow-up. Comparison of groups 1 and 2 revealed no significant differences in all four measurements (RH, RI, RT, and UV) at one, two, three, and twelve months after percutaneous pinning (all *P* ≥ 0.136) (Table 3). However, it seemed that there was a trend in both groups toward recurrent collapse in the first two months after fixation than thereafter (Figure 1). All fractures in this cohort were clinically and radiographically healed at the time of follow-up examinations three months after surgery. Further analyses on the monthly changes of radiographic measurements after fracture fixation revealed that the fixation technique used in group 1 patients produced less recurrent fracture collapse in month-1 RT (3.06 ± 6.51 degrees versus 6.43 ± 6.02 degrees, *P* = 0.028) and month-2 RT (0.63 ± 1.89 degrees versus 1.75 ± 2.57 degrees, *P* = 0.047) than that seen in group 2 patients. There were no significant differences in the monthly changes of other radiographic measurements between the two groups (all *P* ≥ 0.144) (Table 4). When the final loss of alignment after fracture fixation was compared between the two groups, group 1 patients had significantly less loss of alignment in RT (3.78 ± 7.09 degrees versus 8.39 ± 6.74 degrees, *P* = 0.006) than group 2 patients. Although there was a trend toward a better maintenance of global alignment in group 1 patients, this was not statistically significant in RH, RI, and UV (all *P* ≥ 0.267) (Table 5 and Figure 2).

## 4. Discussion 

Although there are myriad factors that affect patient satisfaction following a distal radius fracture, the main treatment objectives are (1) to achieve and maintain the reduced anatomic alignment until bone healing; (2) to decrease associated wrist pain; and (3) to encourage early joint motion [1–7, 19]. Conventional pinning (Willenegger) technique remains a key method of percutaneous pinning for displaced distal radius fractures [9]; however, the reliability of achievement and maintenance of the reduced position until bone healing is always a concern [13, 14, 16, 17]. Kapandji advocated and popularized the technique of intrafocal manipulation and pinning with improving stability and allowing unprotected early motion immediately after surgery [10, 11]. With this technique, Epinette et al. reported 83% of excellent and good results in their case series [20]. Although promising, others found in their prospective studies that there was an increased frequency of pin related complications after Kapandji pinning when compared to that after Willenegger pinning [21–24]. We therefore modified the original Kapandji technique (intrafocal manipulation and transfocal, not intrafocal, fixation) in our clinical practice to decrease skin indentation, tethering, and/or other intrafocal-pin related complications. Our results in this study revealed that there was a trend toward a decrease in frequency of complications in patients treated by the modified Kapandji technique compared with those treated by the Willenegger pinning method, although the differences were not statistically significant.

Our study showed that early fracture collapse occurred in patients with a displaced extra-articular distal radius fracture treated by percutaneous pinning and early mobilization. However, the final radiographic results of the two methods which we studied were both described as acceptable in terms of the four radiographic measurements according to the criteria of Graham [7]. Compared with the Willenegger method, the modified Kapandji technique achieved a less but adequate initial restoration of RH/RI and provided a better maintenance of the reduced position until bone healing. The key of the modified Kapandji technique for improved maintenance of the reduced anatomic alignment is supposed to be the combination of intrafocal manipulation and cross-pin transfocal fixation. Intrafocal manipulation provides a sufficient realignment and a good contact at the fracture surface while avoiding overtraction of the fracture site and undue stress concentration in the implant [25]. Cross-pin transfocal fixation provides a great resistance to gross rotational displacement and prevents the intrafocal-pin related complications such as implant impingement pain, stiffness, CRPS, infection, loosening, or nonunion [26, 27]. The postoperative hand care regimen used in this study permits early wrist and hand mobilization, which is now moderately recommended by the AAOS [8].

Among the radiographic measurements, the palmar RT was the most difficult to achieve and to maintain when repositioning and pinning the fractured fragments and realigning the radial geometry. Normal palmar RT should average about 11 degrees [18]. However, none of the two techniques we used in this study was reliable in restoring the long term normal palmar RT even in the presence of conventionally acceptable radiographic results. The techniques of closed reduction usually depend on ligamentotaxis to restore the RT. Bartosh and Saldana found that when traction is applied across the wrist, the palmar radiocarpal ligaments, which are short and tough, tighten first and then pull on the distal fragment before the thinner dorsal radiocarpal ligaments exert any traction, thus limiting the ability of closed reduction techniques to restore the normal palmar RT [16, 28]. It is interesting that the intrafocal reduction technique did not achieve a better restoration of the RT than that assisted by Chinese finger traps. Even though a higher percentage of AO/OTA type 23-A3 fractures in group 1 patients (76% versus 60%, *P* = 0.160) may explain this observation, we recommend that further refinement of surgical techniques and postoperative hand care program may be necessary in future clinical practices and studies.

Several limitations of this study are noted. First, a weakness is its retrospective nature with the inherent limitations of such a study design. Second, there is lack of functional outcome measurements in this investigation. For treatment of displaced extra-articular fractures of the distal radius, percutaneous pinning techniques have been well-documented surgical methods with distinct correlations between the functional results and the radiographic outcome measures [1–7, 19]. The purpose of this retrospective study was therefore to determine the effects of different methods on the maintenance of the reduced position until bone healing, leaving functional outcome considerations aside. Finally, only AO/OTA type 23-A2.2 or 23-A3 fractures were included. Catalano et al. had found that the final functional results do not correlate with the magnitude of the residual step and gap displacement at the time of fracture healing [29]. In order to minimize systemic errors and bias, we excluded all intra-articular fractures of the distal radius in the current work.

## 5. Conclusions

For displaced extra-articular fractures of the distal radius treated with percutaneous pinning, our time course study revealed that early fracture collapse with loss of the reduced RT occurred. Besides, among the 4 measurements, the normal RT was the most difficult to be achieved when repositioning and pinning the fractured fragments. The modified Kapandji technique provided a superior ability to maintain the reduced position until bone healing over the Willenegger pinning. The intrafocal manipulation may create a good stable contact at the fracture surface, whereas the cross-pin transfocal fixation may provide a great resistance to displacement and may prevent intrafocal-pin related complications. Even though the results are promising, we recommended that refinement of surgical techniques and postoperative hand care program may be necessary to fulfill the treatment objectives of stable surgical fixation and early joint motion.

## Figures and Tables

**Table 1:** Group comparison of patient characteristics and treatment complications.

Variables	Group 1	Group 2	*P* values^†^
	(*n* = 25)	(*n* = 60)	
Mean age (yrs, range)	54.7 (20, 80)	59.9 (24, 76)	0.095
Gender (*n*, %)			0.235^‡^
Male	8 (32)	12 (20)	
Female	17 (68)	48 (80)	
Side (*n*, %)			1.000^‡^
Right	10 (40)	24 (40)	
Left	15 (60)	36 (60)	
AO fracture type (*n*, %)			0.160^‡^
A2.2	6 (24)	24 (40)	
A3	19 (76)	36 (60)	
Complications (*n*, %)			
Nonunion	0 (0)	0 (0)	1.000^‡^
CRPS^a^	1 (4)	1 (1.7)	0.518^‡^
Infection	2 (8)	7 (11.7)	0.665^‡^
Loosening	4 (16)	13 (21.7)	0.552^‡^

^†^Wilcoxon rank sum test, unless otherwise stated.

^‡^Fischer's exact or chi-squared tests as applicable.

^a^CRPS: complex regional pain syndrome.

**Table 2:** Group comparison of radiographic parameters before/after fracture reduction.

Variables	Group 1	Group 2	*P* values^†^
	(*n* = 25)	(*n* = 60)	
Pre-op (mean, SD)			
RH^a^ (mm)	9.63 (2.84)	8.76 (2.31)	0.143
RI^b^ (degree)	20.00 (5.37)	18.63 (4.43)	0.228
RT^c^ (degree)	−17.64 (12.81)	−19.01 (9.81)	0.757
UV^d^ (mm)	3.25 (3.04)	3.74 (2.24)	0.617
Post-op (mean, SD)			
RH (mm)	11.82 (2.32)	12.13 (1.80)	0.510
RI (degree)	23.63 (4.05)	24.36 (2.60)	0.323
RT (degree)	2.06 (5.78)	3.78 (4.35)	0.136
UV (mm)	1.36 (2.70)	1.37 (1.65)	0.996
Δ^e^ Post-pre-op (mean, SD)			
RH (mm)	2.20 (1.36)	3.38 (2.06)	0.010*
RI (degree)	3.64 (2.59)	5.73 (3.86)	0.015*
RT (degree)	19.70 (11.07)	22.80 (10.56)	0.229
UV (mm)	−2.07 (2.55)	−2.37 (1.75)	0.536

^†^Wilcoxon rank sum test, unless otherwise stated.

*The difference is significant (*P* < 0.05).

^a^RH: radial height; ^b^RI: radial inclination; ^c^RT: radial tilt; ^d^UV: ulnar variance; ^e^Δ*Y*_2_ − *Y*_1_: change of *Y*.

**Table 3:** Group comparison of radiographic parameters after fracture fixation.

Variables	Group 1	Group 2	*P* values^†^
	(*n* = 25)	(*n* = 60)	
RH^a^ (mm) (mean, SD)			
Post-op	11.82 (2.32)	12.13 (1.80)	0.510
M1^§^	11.10 (2.63)	11.04 (2.15)	0.911
M2	10.86 (2.76)	10.76 (2.22)	0.856
M3	10.82 (2.81)	10.68 (2.20)	0.800
M12	10.84 (2.49)	10.65 (2.18)	0.674
RI^b^ (degree) (mean, SD)			
Post-op	23.63 (4.05)	24.36 (2.60)	0.323
M1	22.65 (4.80)	22.77 (3.79)	0.905
M2	22.48 (4.93)	22.52 (3.80)	0.962
M3	22.52 (5.01)	22.45 (3.86)	0.945
M12	22.48 (4.95)	22.42 (3.85)	0.724
RT^c^ (degree) (mean, SD)			
Post-op	2.06 (5.78)	3.78 (4.35)	0.136
M1	−1.00 (8.62)	−2.65 (8.38)	0.415
M2	−1.63 (9.44)	−4.40 (9.16)	0.212
M3	−1.72 (8.94)	−4.60 (9.04)	0.182
M12	−1.62 (8.93)	−4.58 (8.94)	0.154
UV^d^ (mm) (mean, SD)			
Post-op	1.36 (2.70)	1.37 (1.65)	0.996
M1	2.34 (2.35)	2.73 (2.02)	0.446
M2	2.89 (2.37)	3.08 (2.23)	0.728
M3	3.23 (2.49)	3.21 (2.23)	0.976
M12	3.19 (2.32)	3.24 (2.11)	0.873

^†^Wilcoxon rank sum test, unless otherwise stated.

^a^RH: radial height; ^b^RI: radial inclination; ^c^RT: radial tilt; ^d^UV: ulnar variance.

^§^M*x*: *x* months after the index surgery.

**Table 4:** Group comparison of time-dependent change of radiographic parameters after fracture fixation.

Variables	Group 1	Group 2	*P* values^†^
	(*n* = 25)	(*n* = 60)	
RH^a^ (mm) (mean, SD)			
Δ^e^ Post-M1^§^	0.72 (0.96)	1.10 (1.63)	0.292
Δ M1-M2	0.24 (0.77)	0.28 (0.69)	0.882
Δ M2-M3	0.04 (0.52)	0.08 (0.46)	0.723
RI^b^ (degree) (mean, SD)			
Δ Post-M1	0.98 (2.00)	1.60 (3.02)	0.354
Δ M1-M2	0.17 (1.23)	0.24 (1.10)	0.798
Δ M2-M3	−0.04 (0.79)	0.07 (0.85)	0.557
RT^c^ (degree) (mean, SD)			
Δ Post-M1	3.06 (6.51)	6.43 (6.02)	0.028*
Δ M1-M2	0.63 (1.89)	1.75 (2.57)	0.047*
Δ M2-M3	0.09 (1.34)	0.21 (1.24)	0.547
UV^d^ (mm) (mean, SD)			
Δ Post-M1	−0.98 (1.25)	−1.36 (1.23)	0.237
Δ M1-M2	−0.55 (0.76)	−0.35 (0.65)	0.227
Δ M2-M3	−0.34 (0.78)	−0.14 (0.47)	0.144

^†^Wilcoxon rank sum test, unless otherwise stated.

*The difference is significant (*P* < 0.05).

^a^RH: radial height; ^b^RI: radial inclination; ^c^RT: radial tilt; ^d^UV: ulnar variance; ^e^Δ*Y*_2_ − *Y*_1_: change of *Y*.

^§^M*x*: *x* months after the index surgery.

**Table 5:** Group comparison of the final loss of alignment after fracture healing.

Variables	Group 1	Group 2	*P* values^†^
	(*n* = 25)	(*n* = 60)	
Δ^e^ Post-M3^§^ (mean, SD)			
RH^a^ (mm)	1.00 (1.31)	1.45 (1.67)	0.305
RI^b^ (degree)	1.11 (2.46)	1.91 (3.21)	0.267
RT^c^ (degree)	3.78 (7.09)	8.39 (6.74)	0.006*
UV^d^ (mm)	−1.85 (1.44)	−1.85 (1.44)	0.958

^†^Wilcoxon rank sum test, unless otherwise stated.

*The difference is significant (*P* < 0.05).

^a^RH: radial height; ^b^RI: radial inclination; ^c^RT: radial tilt; ^d^UV: ulnar variance; ^e^Δ*Y*_2_ − *Y*_1_: change of *Y*.

^§^M*x*: *x* months after the index surgery.
